# Correlates of Treatment Outcomes and Drug Resistance among Pulmonary Tuberculosis Patients Attending Tertiary Care Hospitals of Kolkata, India

**DOI:** 10.1371/journal.pone.0109563

**Published:** 2014-10-07

**Authors:** Avranil Goswami, Urmita Chakraborty, Tanmay Mahapatra, Sanchita Mahapatra, Tapajyoti Mukherjee, Shibali Das, Aritra Das, Subir Kumar Dey, Sudhin Ray, Basudev Bhattacharya, Nishith Kumar Pal

**Affiliations:** 1 Institute of Postgraduate Medical Education & Research, Kolkata, India; 2 Jonathan and Karin Fielding School of Public Health, University of California Los Angeles, Los Angeles, California, United States of America; 3 Burdwan Medical College & Hospital, Burdwan, India; 4 Bose Institute, Kolkata, India; 5 Calcutta National Medical College, Kolkata, India; 6 SSKM Hospital, Kolkata, India; Universidad Nacional de La Plata, Argentina

## Abstract

**Background:**

Worldwide highest number of new pulmonary tuberculosis (PTB) cases, was reported from India in 2012. Adverse treatment outcomes and emergence of drug resistance further complicated the prevailing scenario owing to increased duration, cost and toxicity associated with the treatment of drug-resistant cases. Hence to reinforce India’s fight against TB, identification of the correlates of adverse treatment outcomes and drug resistance, seemed critical.

**Methods:**

To estimate the associations between diagnostic findings, patient types (based on treatment outcomes), drug resistance and socio-demographic characteristics of PTB patients, a cross-sectional study was conducted in two tertiary-care hospitals in Kolkata between April 2010 and March 2013. Altogether, 350 consenting *Mycobacterium tuberculosis* sputum-culture positive PTB patients were interviewed about their socio-demographic background, evaluated regarding their X-ray findings (minimal/moderately advanced/far advanced/cavities), sputum-smear positivity, and treatment history/outcomes (new/defaulter/relapse/treatment-failure cases). Multiple-allele-specific polymerase chain reaction (MAS-PCR) was conducted to diagnose drug resistance.

**Results:**

Among all participants, 31.43% were newly diagnosed, while 44%, 15.43% and 9.14% patients fell into the categories of relapsed, defaulters and treatment-failures, respectively. 12.29% were multi-drug-resistant (MDR: resistant to at least isoniazid and rifampicin), 57.71% had non-MDR two-drug resistance and 12% had single-drug resistance. Subjects with higher BMI had lower odds of being a relapse/defaulter/treatment failure case while females were more likely to be defaulters and older age-groups had more relapse. Elderly, females, unmarried, those with low BMI and higher grade of sputum-smear positivity were more likely to have advanced X-ray features. Higher grade of sputum-smear positivity and advanced chest X-ray findings were associated with relapse/treatment-failures. Elderly, unmarried, relapse/defaulter/treatment-failure cases had higher odds and those with higher BMI and moderately/far advanced X-ray findings had lower odds of having MDR/non-MDR two-drug resistant PTB.

**Conclusion:**

Targeted intervention and appropriate counseling are needed urgently to prevent adverse treatment outcomes and development of drug resistance among PTB patients in Kolkata.

## Introduction

Tuberculosis (TB) continues to be a major public health problem and is currently the second largest infectious cause of death worldwide. [1] As per World Health Organization’s (WHO) estimate, in 2012, approximately 8.6 million people developed TB and 1.3 million died from it. [1] About 58% of these new infections were reported from Asian countries, with India being the largest contributor of incident TB infections in the world (176 per 100,000 population), accounting for 26% of the total global cases in 2012. [1].

WHO-assisted Directly Observed Treatment-Short course (DOTS) program in India not only serves more than 100,000 patients per month but also holds the distinction of being the largest and fastest expanding TB control program in the world.[2]–[5] However, despite the significant strides made by this program, annual incidence of TB in this country still hovers above two million cases, and on an average, two TB-related deaths are reckoned to occur in every three minutes. [4], [5].

Since the anti-tubercular drugs (ATD) have become available, improper regimen, indiscriminate usage and less than optimal adherence have undermined the potential benefits - largely by facilitating the emergence of drug-resistant strains, particularly the multi-drug resistant (MDR) variety. [6], [7] MDR-TB strains are distinguished by their resistance to isoniazid (INH) and rifampicin (RIF), two most potent first line ATDs, but not necessarily to other ATDs. [8] Globally, an estimated 3.6% of new (450,000) and 20.2% of previously-treated TB cases were diagnosed with MDR-TB in 2012. [1] Expectedly, patients with prior exposure to anti-TB therapy turned out to be the ones more vulnerable to developing drug resistance, [9] but it was perturbing to note that even the newly diagnosed cases had considerable propensity of having MDR, with possible contributing factors being spontaneous mutation and transmission of resistant strains from others patients harboring MDR-TB. [10], [11].

Rapid emergence of drug resistance, particularly MDR constitutes a major threat to TB control in India. [7], [12], [13] Data on drug resistance pattern prevailing in the community are scarcely available, and there exists considerable inconsistency among the published findings. [6], [10], [14] A 2012 country-level estimate by WHO reported India’s burden of MDR-TB to be moderately high (about 2.2% of new TB cases and 15% of retreatment cases), [1] however, there was substantial heterogeneity across reviewed studies.

The impact of MDR-TB, in terms of disease containment efforts and economic burden, on the public health infrastructure of a developing nation like India is enormous. Some of the public health challenges associated with it are: expensive laboratory procedures for diagnosis, limited number and capacity of testing facilities, long duration of treatment regime, requirement of multiple expensive, less potent, more toxic and relatively less available second line anti-TB drugs, shortage of trained staff and facilities for treatment and proper monitoring of the infected. [1], [12], [13].

The above shortcomings accompanying MDR-TB program become more glaring while addressing the problem of pulmonary TB (PTB) cases, owing to associated clinical severity. Constraints associated with the diagnosis and treatment of MDR-PTB thus call for efforts to identify the socio-demographic and clinical correlates of drug resistant PTB and poorer treatment outcomes. It is vital to take into consideration such predictors, especially the less resource intensive ones, in order to design and implement effective intervention strategies for minimizing the potential of emergence of resistant cases and improving treatment outcomes (e.g. reduction in number of relapses, defaulters and treatment-failures). In view of the above, a cross-sectional study was conducted to understand the interrelationship between diagnostic features (radiological and microscopic), patient type and drug resistance patterns among PTB patients.

## Methodology

### Ethics Statement

The Institutional Ethics Committee of the Institute of Post-Graduate Medical Education and Research (IPGMER), Kolkata, India approved the study content and procedures.

Written informed consents were collected from each participant before the interview and sample collection. Subjects were free to decline participation without any consequences towards their treatments.

### Recruitment

Current study was conducted in the out and in-patient departments of Chest Medicine in two tertiary care hospitals (the DOTS clinic of SSKM Hospital and Chest clinic of Calcutta National Medical College) of Kolkata, a populous city of eastern India. Consenting sputum-smear-positive PTB patients (according WHO criteria and standard guidelines), [1], [15] were recruited for the study during their first attendance between April 2010 and March 2013. Subjects were excluded from the analyses if they later turned out to be culture-negative for Mycobacterium, had infection with Mycobacterium other than *M. tuberculosis* or respective cultures got contaminated. As the parameter values regarding the variables of interest were not available in the study area, prior calculation of the required sample size was not possible. Hence we decided to recruit all the eligible cases identified during the study period.

### Data Collection

Socio-demographic information such as age (<20/20–45/>45 years), gender (male/female), marital status (unmarried/married), socio-economic status (high/middle/low income group determined based on per capita monthly income, education and occupation of the head of the household using Kuppuswamy’s Socio-Economic Status Scale) and district of residence (Kolkata/24 Parganas South/North) were collected through face-to-face interviews, whereas body mass index (Body weight(kg)/Height^2^(mt)) and blood hemoglobin (gm/dl) levels were measured by trained medical/paramedical staff. Based on the clinical history of diagnosis and prior treatment outcomes (if any), recruited PTB cases were classified (patient types) into new/relapse/defaulter/treatment failure according to WHO criteria. [1].

### Chest X-ray and Sputum-smear Examination

Chest radiographic results of the participants, as evaluated by clinicians, were categorized into minimal/moderately advanced/far advanced/having cavities. [15], [16] As per the WHO and Government of India collaborative guidelines, grade of sputum-smear positivity was classified into scanty/single/double/triple positive status based on microscopic findings. [17].

### Laboratory testing

Morning sputum samples were collected from each patient and processed by digestion and decontamination using the *N*-acetyl-L-cysteine (NALC)/sodium hydroxide (NaOH) method. [7] The resuspended sediment from the decontaminated pellet was then used for Ziehl-Neelsen (ZN) staining, inoculation on Lowenstein-Jensen (LJ) solid and Middlebrook 7H9 liquid media. [7], [18].

Biochemical tests (niacin test, catalase test, nitrate reduction and aryl sulfatase) were next performed to identify *M. tuberculosis* among the Mycobacterial isolates. [13] The positive cultures were also subjected to extraction of genomic DNA following the phenol-chloroform method. [19] Molecular confirmation was carried out by multiplex-PCR [20] for amplification of hsp *65, dnaj, is6110* genes specific to *M. tuberculosis*.

### Detection of drug resistance pattern

For rapid detection of MDR isolates, multiple-allele-specific PCR (MAS-PCR) was conducted using allele-specific primers. [21] For detection of Isoniazid (INH), Rifampicin (RIF) or Ethambutol (EMB) resistance, amplifications of *katG* gene and promoter region of mabA-inhA, *rpoB* gene, *embB* gene were carried out, respectively. The amplified products were visualized in a 2.5% metaphor gel under a UV GEL DOC (BIO-RAD). Based on the results, participating TB cases were classified into those infected with MDR/Non-MDR two drug/Single drug resistant or susceptible strains of *M. tuberculosis*.

All the radiological (chest X-ray) and laboratory investigation results (sputum smear examination, MAS-PCR and biochemical tests) were duly double checked by a team of independent experts in the respective departments.

### Data analyses

Descriptive analyses were done to understand the overall distribution (mean/proportion and corresponding 95% confidence intervals (CI)) of the patient characteristics along with drug resistance patterns. Further, stratified distributions of these characteristics across the strata of drug resistance were examined. Bivariate and multivariate logistic regression analyses [odds ratio (OR), adjusted odds ratio (AOR) and corresponding 95%CIs] were next conducted, respectively, to determine crude (OR) and adjusted (AOR: each predictor adjusted for all others) association between study variables. As the dependent variables (patient types, X-ray features and drug resistance patterns) had more than two categories, multinomial logistic regressions were used. [22] All statistical analyses were conducted using SAS version 9.3.

## Results

**Figure 1:**
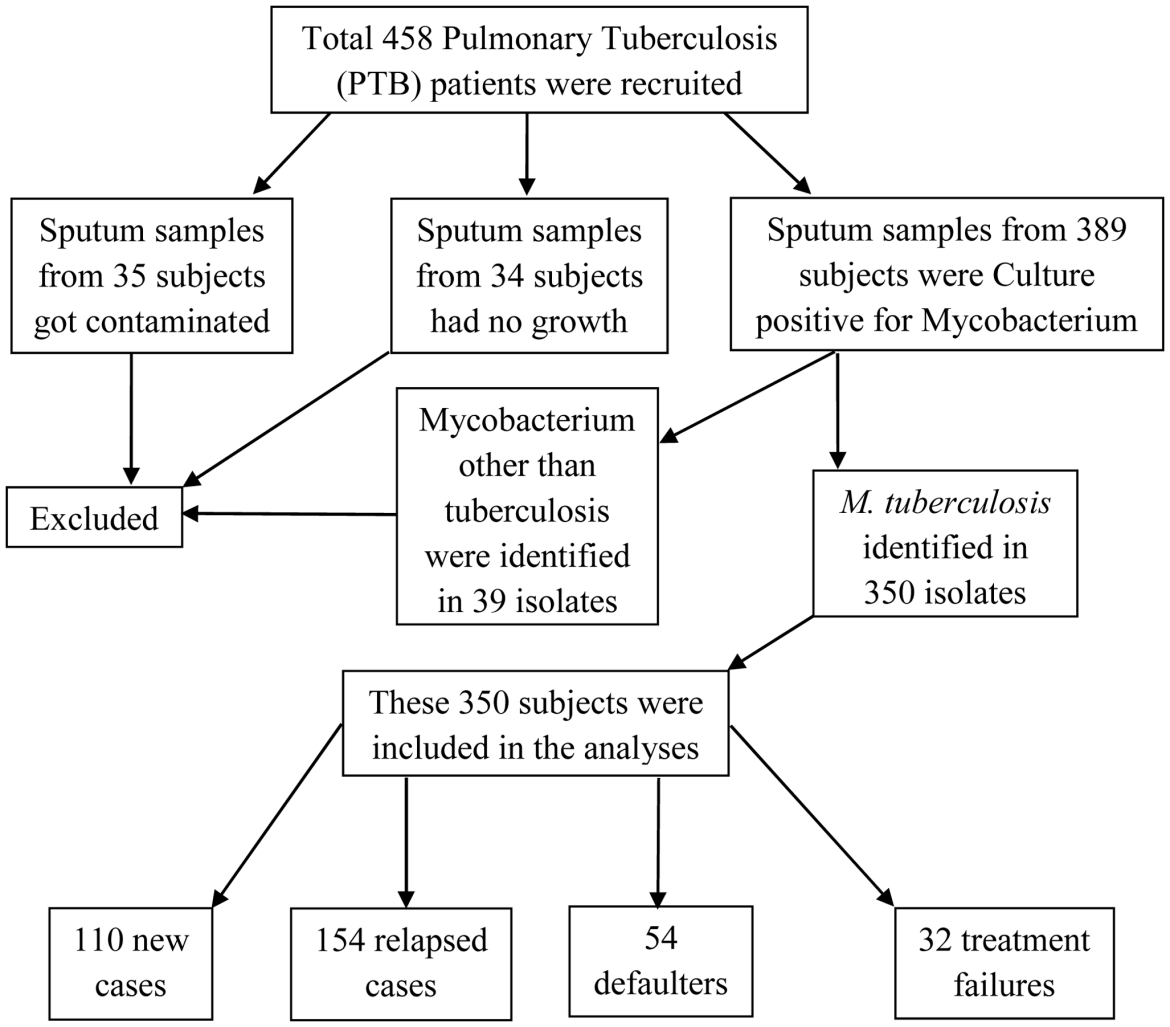
Flow chart depicting the process of recruitment of the study participants.

Among the sputum samples collected from 458 recruited PTB cases, 35 (7.6%) got contaminated and 34 (7.4%) showed no growth. Biochemical tests and multiplex-PCR of the remaining 389 culture-positive isolates identified 350 samples positive for *M. tuberculosis* while rest 39 belonged to different Mycobacterium species. (Figure 1).

Thus, altogether 350 PTB cases having *M. tuberculosis* positive sputum were included in the final analyses. Majority of them were male (71.43%), aged between 20–45 years (72.29%), married (78.57%), from low-income group (86.57%) and residents of Kolkata (80.29%). Mean BMI of the subjects was 19.24 kg/m^2^ and mean blood hemoglobin level was 10.68 gm/dl. While 31.43% (95%CI: 26.54–36.32) of the TB cases were newly diagnosed, the proportions of relapse cases, defaulters and treatment-failures were 44.00% (95%CI: 38.77–49.23), 15.43% (95%CI: 11.63–19.23) and 9.14% (95%CI: 6.11–12.18), respectively. X-Ray findings in 37.71% patients fell into the category of moderately advanced, 17.14% had far advanced X-Ray features and 9.14% had evidence of cavities. Single-positive sputum-smears were obtained from 47.14% cases, whereas double-positive and triple-positive results, respectively, were seen in 44.86% and 8.00% of patients. Among the included sputum-smear positive subjects, 12.29% (95%CI: 8.83–15.74) had MDR-TB, 57.71% (95%CI: 52.51–62.92) had non-MDR two drug resistance, while 12% (95%CI: 8.58–15.42) had resistance to any one of the first line of drugs (5.71%, 4.29% and 2.00% to only INH, Ethambutol and Rifampicin, respectively). Isolates obtained from only 18.00% (95%CI: 13.96–22.04) patients were susceptible to all three first line ATDs. (Table 1).

**Table 1:** Distribution of socio-demographics, patient types, physical parameters, microscopic features, X-ray findings and drug resistance patterns among PTB patients in tertiary care hospitals of Kolkata, India, 2010–2013 (N = 350).

Continuous Variables		n	Mean (95% CI)
Body mass index (kg/m^2^)		350	19.24 (19.07–19.41)
Blood hemoglobin level (gm/dl)		350	10.68 (10.52–10.85)
**Categorical Variables**	**Categories**	**n**	**Percentage (95% CI)**
Gender	Male	250	71.43 (66.67–76.18)
	Female	100	28.57 (23.82–33.33)
Age	<20 Yrs	48	13.71 (10.09–17.35)
	20–45 Yrs	253	72.29 (67.57–77.00)
	>45 Yrs	49	14.00 (10.35–17.65)
Marital Status	Married	275	78.57 (74.25–82.89)
	Unmarried	75	21.43 (17.11–25.75)
Socio-economic status	LIG	303	86.57 (82.98–90.16)
	MIG	45	12.86 (9.33–16.38)
	HIG	2	0.57 (0.00–1.37)
Resident of	North 24 Parganas	13	3.71 (1.72–5.71)
	South 24 Parganas	56	16.00 (12.14–19.85)
	Kolkata	281	80.29 (76.10–84.47)
Patient type	New Infection	110	31.43 (26.54–36.32)
	Relapsed	154	44.00 (38.77–49.23)
	Defaulter	54	15.43 (11.63–19.23)
	Treatment failure	32	9.14 (6.11–12.18)
Sputum smear grade	Scanty	0	0
	Single positive	165	47.14 (41.89–52.40)
	Double positive	157	44.86 (39.62–50.09)
	Triple positive	28	8.00 (5.14–10.86)
X Ray Findings	Minimal	126	36.00 (30.95–41.05)
	Moderately advanced	132	37.71 (32.61–42.82)
	Far advanced	60	17.14 (13.18–21.11)
	Cavity	32	9.14 (6.11–12.18)
Multiple allele specific PCR for detection of drug resistance	Multi-drug resistance	43	12.29 (8.83–15.74)
	Non-MDR two drug resistance	202	57.71 (52.51–62.92)
	Only Isoniazide resistance*	20	5.71 (3.27–8.16)
	Only Rifampicin resistance*	15	4.29 (2.15–6.42)
	Only Ethambutol resistance*	7	2.00 (0.53–3.47)
	Susceptible	63	18.00 (13.96–22.04)

N = Total number of subjects in the study; n = Number of subjects in different categories.

CI = Confidence interval; LIG = Low-income group; MIG = Middle-income group; HIG = High-income group *Total number of single drug resistant cases = 42.

Distribution of the socio-demographic characteristics along with clinical, radiological and laboratory parameters across the strata of drug resistance pattern is presented in Table 2. Results of bivariate and multivariate (each predictor adjusted for all others) regression analyses (to determine association between patient types and their physical/socio-demographic/microscopic/X-ray features) are presented in Table 3. Patients with higher mean BMI had lower odds (reference: new infection) of being a relapse [AOR = 0.06(0.03–0.11)]/defaulter [AOR = 0.45(0.28–0.74)]/treatment failure [AOR = <0.01(<0.01–0.01)] TB case; while those with higher hemoglobin levels had twice the odds (compared to being a new infection) of belonging to relapse [AOR = 1.94(1.22–3.07)] or defaulter [AOR = 4.00(2.55–6.26)] category. Female PTB cases, as against males, had 2.6 times higher odds of being a defaulter [AOR = 3.66(1.33–10.06)] than being a new infection case. Older age groups, compared to those aged <20 years, had significantly higher odds [20–45 years: AOR = 10.44(1.69–64.46) and >45 years: AOR = 8.95(1.05–76.41)] of having relapse than having new infection. Patients with worse categories of sputum positivity (vs. single positive sputum) and poorer X-ray features (vs. minimal advanced) were detected to have increased odds of having relapse/treatment-failure, although the associations did not reach levels of statistical significance, possibly due to lack of power. (Table 3).

**Table 2:** Distribution of drug resistance patterns across the strata of socio-demographics, patient type, X-ray features and microscopic findings among PTB patients in tertiary care hospitals of Kolkata, India, 2010–2013 (N = 350).

		Susceptible		Single drug resistance		Non-MDR two drug resistance		Multi-drug resistance (MDR)	
Continuous Variables		n	Mean (95% CI)	n	Mean (95% CI)	n	Mean (95% CI)	n	Mean (95% CI)
Body mass index (kg/m^2^)		63	19.99(19.63–20.35)	42	19.74(19.25–20.22)	202	18.99(18.78–19.20)	43	18.87(18.37–19.36)
Blood hemoglobin level (gm/dl)		63	10.40(9.98–10.81)	42	10.43(9.89–10.97)	202	10.85(10.64–11.06)	43	10.58(10.12–11.05)
**Categorical variables**		**Susceptible**		**Single drug resistant**		**Non-MDR two drug resistant**		**MDR**	
**Variables**	**Categories**	**n**	**Percentage (95% CI)**	**n**	**Percentage (95% CI)**	**n**	**Percentage (95% CI)**	**n**	**Percentage (95% CI)**
Gender	Male	44	69.84(58.19–81.49)	31	73.81(59.94–87.68)	143	70.80(64.47–77.12)	32	74.42(60.83–88.01)
	Female	19	30.16(18.51–41.81)	11	26.19(12.32–40.06)	59	29.21(22.88–35.53)	11	25.58(11.99–39.16)
Age	<20 Yrs	15	23.81(12.99–34.62)	6	14.29(3.25–25.32)	23	11.39(6.97–15.8)	4	9.30(0.26–18.35)
	20–45 Yrs	44	69.84(58.19–81.49)	28	66.67(51.80–81.53)	150	74.26(68.18–80.34)	31	72.09(58.12–86.06)
	>45 Yrs	4	6.35(0.15–12.54)	8	19.05(6.66–31.43)	29	14.36(9.48–19.23)	8	18.60(6.49–30.72)
Marital Status	Married	45	71.43(59.96–82.90)	33	78.57(65.63–91.51)	162	80.20(74.66–85.74)	35	81.40(69.28–93.51)
	Unmarried	18	28.57(17.10–40.04)	9	21.43(8.49–34.37)	40	19.80(14.26–25.34)	8	18.60(6.49–30.72)
Socio-economic status	LIG	52	82.54(72.90–92.18)	35	83.33(71.58–95.09)	179	88.61(84.20–93.03)	37	86.05(75.26–96.84)
	MIG	11	17.46(7.82–27.09)	6	14.29(3.25–25.32)	22	10.89(6.56–15.22)	6	13.95(3.16–24.74)
	HIG	–	–	1	2.381(0–7.19)	1	0.50(0–1.47)	–	–
Resident of	North 24 Parganas	2	3.17(0.00–7.63)	4	9.52(0.27–18.78)	7	3.47(0.92–6.00)	–	–
	South 24 Parganas	5	7.94(1.07–14.80)	9	21.43(8.49–34.37)	37	18.32(12.93–23.70)	5	11.63(1.65–21.61)
	Kolkata	56	88.89(80.91–96.87)	29	69.05(54.47–83.63)	158	78.22(72.48–83.96)	38	88.37(78.39–98.35)
Patient type	New Infection	44	69.84(58.19–81.49)	23	54.76(39.06–70.46)	36	17.82(12.50–23.14)	7	16.28(4.78–27.78)
	Relapsed	13	20.63(10.36–30.91)	15	35.71(20.60–50.83)	106	52.48(45.53–59.42)	20	46.51(30.98–62.04)
	Defaulter	5	7.94(1.07–14.80)	3	7.14(0–15.27)	38	18.81(13.38–24.25)	8	18.60(6.49–30.72)
	Treatment failure	1	1.59(0.00–4.76)	1	2.38(0–7.19)	22	10.89(6.56–15.22)	8	18.60(6.49–30.72)
Sputum smear grade	Scanty	0	0	0	0	0	0	0	0
	Single positive	34	53.97(41.31–66.62)	12	28.57(14.32–42.82)	99	49.00(42.06–55.96)	20	46.51(30.98–62.04)
	Double positive	27	42.86(30.29–55.42)	28	66.67(51.80–81.53)	81	40.10(33.28–46.91)	21	48.84(33.27–64.40)
	Triple positive	2	3.17(0–7.63)	2	4.76(0–11.48)	22	10.89(6.56–15.22)	2	4.65(0–11.21)
X- Ray Findings	Minimal	21	33.33(21.37–45.30)	11	26.19(12.32–40.06)	76	37.62(30.89–44.36)	18	41.86(26.50–57.22)
	Moderately advanced	27	42.86(30.30–55.42)	18	42.86(27.25–58.47)	74	36.63(29.93–43.33)	13	30.23(15.93–44.53)
	Far advanced	12	19.05(9.08–29.02)	9	21.43(8.49–34.37)	29	14.36(9.48–19.23)	10	23.26(10.10–36.41)
	Cavity	3	4.76(0–10.17)	4	9.52(0.27–18.78)	23	11.39(6.97–15.80)	2	4.65(0–11.21)

N = Total number of subjects in the study; n = Number of subjects in different categories.

CI = Confidence interval; LIG = Low-income group; MIG = Middle-income group; HIG = High-income group.

**Table 3:** Association between patient type and their physical, socio-demographic, microscopic and X-ray features among PTB patients in tertiary care hospitals of Kolkata, India, 2010–2013 (N = 350).

			Patient type(ref: New infection)					
			Relapsed		Defaulter		Treatment failure	
Continuous Variables			OR(95%Cl)	*p*	OR(95%Cl)	*p*	OR(95%Cl)	*p*
Body mass index (kg/m^2^)		UOR	0.06(0.04–0.11)	<.001	0.44(0.30–0.66)	<.001	<0.01(<0.01–0.01)	<.001
		AOR	**0.06(0.03–0.11)**	**<.001**	**0.45(0.28–0.74)**	**0.002**	**<0.01(<0.01–0.01)**	**<.001**
Blood hemoglobin level (gm/dl)		UOR	2.58(1.98–3.35)	<.001	3.33(2.32–4.79)	<.001	1.47(1.03–2.10)	0.035
		AOR	**1.94(1.22–3.07)**	**0.005**	**4.00(2.55–6.26)**	**<.001**	0.67(0.29–1.57)	0.354
**Categorical variables**			**Relapsed**		**Defaulter**		**TF**	
**Variables**	**Categories**		**OR(95%Cl)**	***p***	**OR(95%Cl)**	***p***	**OR(95%Cl)**	***p***
Gender (ref: male)	Female	UOR	0.71(0.41–1.24)	0.228	1.27(0.63–2.53)	0.503	1.40(0.61–3.19)	0.423
		AOR	1.18(0.38–3.61)	0.779	**3.66(1.33–10.06)**	**0.012**	1.70(0.32–9.01)	0.535
Age (ref:<20 yrs)	20–45 yrs	UOR	2.50(1.21–5.16)	0.013	1.57(0.65–3.83)	0.319	2.35(0.65–8.55)	0.194
		AOR	**10.44(1.6964.46)**	**0.012**	2.80(0.59–13.32)	0.195	65.61(2.78–>99.99)	0.010
	>45 yrs	UOR	3.56(1.37–9.24)	0.009	1.20(0.32–4.47)	0.788	3.83(0.81–18.09)	0.090
		AOR	**8.95(1.05–76.41)**	**0.045**	1.76(0.25–12.71)	0.574	77.02(1.90–>99.99)	0.022
Marital status (ref: Married)	Unmarried	UOR	0.33(0.18–0.60)	0.001	0.40(0.17–0.89)	0.026	0.55(0.22–1.40)	0.210
		AOR	0.99(0.19–5.21)	0.990	0.58(0.14–2.45)	0.455	5.27(0.36–76.93)	0.224
SES (ref: LIG)	MIG	UOR	0.52(0.24–1.09)	0.081	0.89(0.36–2.19)	0.799	0.98(0.33–2.90)	0.975
		AOR	0.46(0.13–1.72)	0.251	0.69(0.23–2.10)	0.515	1.57(0.19–12.99)	0.678
	HIG	UOR	–	–	–	–	–	–
		AOR	–	–	–	–	–	–
Resident of (ref: Kolkata)	North 24 Parganas	UOR	0.23(0.05–1.19)	0.079	1.35(0.36–5.03)	0.656	0.54(0.06–4.66)	0.573
		AOR	0.14(0.01–2.19)	0.160	1.55(0.31–7.76)	0.593	0.05(0.01–2.60)	0.139
	South 24 Parganas	UOR	1.16(0.60–2.24)	0.669	0.83(0.32–2.16)	0.707	0.76(0.24–2.45)	0.643
		AOR	0.88(0.25–3.08)	0.846	0.55(0.15–1.10)	0.366	0.22(0.03–1.90)	0.170
Sputum smear grading (ref: Single positive)	Double positive	UOR	2.55(1.52–4.29)	0.001	1.07(0.54–2.14)	0.840	11.81(3.28–42.53)	0.001
		AOR	1.48(0.47–4.73)	0.505	1.01(0.35–2.90)	0.988	2.23(0.25–19.85)	0.472
	Triple positive	UOR	3.45(1.06–11.26)	0.040	1.05(0.18–6.00)	0.960	57.49(11.18–295.51)	<.001
		AOR	0.84(0.07–10.56)	0.892	1.05(0.06–17.33)	0.971	0.92(0.03–32.08)	0.963
X-ray (ref: Minimal)	Moderately advanced	UOR	1.69(1.00–2.95)	0.065	1.67(0.82–3.38)	0.157	**12.60(1.54–103.43)**	**0.018**
		AOR	1.15(0.38–3.49)	0.806	1.99(0.78–5.07)	0.152	1.64(0.11–24.83)	0.722
	Far advanced	UOR	2.86(1.29–6.37)	0.010	1.69(0.58–4.96)	0.334	76.35(9.12–639.18)	<.001
		AOR	1.95(0.32–11.91)	0.468	1.66(0.30–9.24)	0.563	9.77(0.44–217.99)	0.150
	Cavity	UOR	8.17(2.29–29.07)	0.001	0.89(0.09–9.03)	0.921	130.65(1.91–>99.99)	<.001
		AOR	4.54(0.41–50.66)	0.220	0.55(0.02–13.24)	0.713	33.93(0.84–>99.99)	0.062

N = Total number of subjects in the study; SES = Socio-economic status; LIG = Low-income group; MIG = Middle-income group; HIG = High-income group; UOR = Unadjusted Odds ratio; AOR = Adjusted Odds ratio; OR = Odds ratio; CI = Confidence interval; *p* = *p* value.

“–” refers to cells for which due to inadequate number of observation valid statistical results (OR, CI, *p* value) could not be determined.

Patients with higher mean BMI had lower odds of having poorer X-ray features [AOR for: moderately advanced = 0.71(0.55–0.91) and far advanced = 0.69(0.49–0.98)], whereas cases diagnosed with double-positive sputum-smear (vs. single-positive sputum) were more likely to have the same [AOR for: moderately advanced = 3.40(1.88–6.14) and far advanced = 88.95(19.72–401.20)], compared to having minimal advanced X-ray features. Female [AOR = 2.28(1.18–4.41, reference: male)], elderly [AOR = 4.13(1.02–16.68, reference: age <20 years)] and unmarried [AOR = 4.39(1.63–11.86, reference: married)] cases were also found to have higher odds of moderately advanced (compared to minimal advanced) X-ray features. (Table 4).

**Table 4:** Association of X-ray findings with physical, socio-demographic and microscopic features among PTB patients in tertiary care hospitals of Kolkata, India, 2010–2013 (N = 350).

			X-Ray findings (ref: Minimal)					
			Moderately advanced		Far advanced		Cavity	
Continuous Variables			OR(95%Cl)	*p*	OR(95%Cl)	*p*	OR(95%Cl)	*p*
Body mass index (kg/m^2^)		UOR	0.71(0.57–0.87)	0.001	0.53(0.40–0.70)	<.0001	0.42(0.29–0.63)	<.0001
		AOR	**0.71(0.55–0.91)**	**0.006**	**0.69(0.49–0.98)**	**0.036**	0.64(0.38–1.05)	0.078
Blood hemoglobin level (gm/dl)		UOR	0.97(0.79–1.19)	0.757	0.96(0.74–1.24)	0.737	1.31(0.93–1.86)	0.119
		AOR	1.01(0.78–1.31)	0.926	0.89(0.62–1.28)	0.537	1.41(0.85–2.37)	0.187
**Categorical variables**			**Moderately advanced**		**Far advanced**		**Cavity**	
**Variables**	**Categories**		**OR(95%Cl)**	***p***	**OR(95%Cl)**	***p***	**OR(95%Cl)**	***p***
Gender (ref:Male)	Female	UOR	1.63(0.93–2.85)	0.085	1.88(0.96–3.71)	0.066	1.37(0.57–3.29)	0.482
		AOR	**2.28(1.18–4.41)**	**0.015**	2.37(0.93–6.04)	0.071	2.96(0.86–10.23)	0.087
Age (ref:<20 yr)	20–45 yr	UOR	0.92(0.45–1.90)	0.832	0.60(0.26–1.38)	0.232	3.89(0.49–30.85)	0.198
		AOR	2.53(0.80–7.98)	0.113	0.56(0.11–2.87)	0.483	0.67(0.03–15.22)	0.800
	>45 yr	UOR	1.45(0.55–3.80)	0.447	0.76(0.23–2.48)	0.653	**11.77(1.32–104.99)**	**0.027**
		AOR	**4.13(1.02–16.68)**	**0.047**	0.66(0.09–4.92)	0.685	1.71(0.06–48.71)	0.752
Marital status (ref: Married)	Unmarried	UOR	1.84(1.01–3.34)	0.045	1.58(0.75–3.31)	0.231	0.15(0.02–1.18)	0.071
		AOR	**4.39(1.63–11.86)**	**0.004**	1.16(0.26–5.10)	0.845	0.12(0.01–2.43)	0.166
SES (ref:LIG)	MIG	UOR	1.25(0.59–2.64)	0.555	1.05(0.40–2.75)	0.925	1.90(0.67–5.44)	0.230
		AOR	1.12(0.48–2.63)	0.786	1.01(0.31–3.33)	0.982	2.27(0.53–9.80)	0.271
	HIG	UOR	–	–	–	–	4.44(0.27–73.42)	0.298
		AOR	–	–	–	–	–	–
Resident of (ref: Kolkata)	North 24 Parganas	UOR	2.04(0.60–6.98)	0.257	0.53(0.06–4.87)	0.575	–	–
		AOR	1.85(0.47–7.27)	0.375	0.33(0.03–3.65)	0.365	–	–
	South 24 Parganas	UOR	1.25(0.63–2.46)	0.526	1.18(0.51–2.74)	0.702	1.33(0.48–3.69)	0.580
		AOR	0.98(0.46–2.08)	0.952	0.81(0.28–2.31)	0.695	1.45(0.40–5.26)	0.574
Sputum smear grading (ref: Single positive)	Double positive	UOR	3.97(2.28–6.90)	<.0001	92.31(21.04–405.03)	<.0001	–	–
		AOR	**3.40(1.88–6.14)**	**<.0001**	**88.95(19.72–401.20)**	**<.0001**	–	–
	Triple positive	UOR	–	–	–	–	–	–
		AOR	–	–	–	–	–	–

SES = Socio-economic status; LIG = Low-income group; MIG = Middle-income group; HIG = High- income group; UOR = Unadjusted Odds ratio; AOR = Adjusted Odds ratio; OR = Odds ratio; CI = Confidence interval; *p* = *p* value.

“–” refers to cells for which due to inadequate number of observation valid statistical results (OR, CI, *p* value) could not be determined.

Cases belonging to older age groups, as against those aged <20 years, had higher odds of having MDR (AOR_MDR_) and non-MDR two drug resistant TB (AOR_NM2DR_) than having a TB strain susceptible to all three first-line drugs [for 20–45 years age-group: AOR_MDR_ = 7.30(1.10–48.59) and AOR_NM2DR_ = 4.59(1.16–18.12) while for >45 years, AOR_MDR_ = 30.01(2.70–333.06) and AOR_NM2DR_ = 12.14(1.82–80.92)]. Unmarried cases (vs. married) had more than four times odds of being diagnosed with either AOR_MDR_ or AOR_NM2DR_, compared to being in the all susceptible group [AOR_MDR_ = 4.83(1.10–48.59) and AOR_NM2DR_ = 4.59(0.99–23.66)]. Higher odds of AOR_MDR_ and AOR_NM2DR_ were also observed for relapse/defaulter/treatment-failure groups, as compared to new infection cases [relapse: AOR_MDR_ = 13.84(2.55–75.06) and AOR_NM2DR_ = 12.02(3.51–41.15); defaulter: AOR_MDR_ = 33.01(5.96–182.78) and AOR_NM2DR_ = 16.87(4.77–59.69); treatment-failure: AOR_MDR_ = 63.19(3.63–>99.99) and AOR_NM2DR_ = 31.86(2.66–381.62)], whereas patients with moderately/far advanced X-ray findings (vs. minimal advanced) were less likely to be suffering from either AOR_MDR_ or AOR_NM2DR_ [moderately advanced: AOR_MDR_ = 0.16(0.05–0.53) and AOR_NM2DR_ = 0.27(0.11–0.67); far advanced: AOR_MDR_ = 0.14(0.03–0.68) and AOR_NM2DR_ = 0.14(0.04–0.49)]. (Table 5).

**Table 5:** Association between patient characteristics and drug resistance patterns among PTB patients in tertiary care hospitals of Kolkata, India, 2010–2013 (N = 350).

				Drug resistant status (ref:Susceptible)					
				Single drug resistance		Non-MDR two drug resistance		Multi-drug resistance (MDR)	
Continuous Variables				OR(95%Cl)	*p*	OR(95%Cl)	*p*	OR(95%Cl)	*p*
Body mass index (kg/m^2^)		UOR		0.83(0.59–1.16)	0.279	0.49(0.38–0.63)	<.0001	0.44(0.31–0.63)	<.0001
		AOR		1.18(0.66–2.13)	0.572	0.73(0.46–1.15)	0.171	0.62(0.34–1.15)	0.130
Blood hemoglobin level (gm/dl)		UOR		1.02(0.74–1.41)	0.896	1.38(1.08–1.75)	0.009	1.14(0.82–1.57)	0.435
		AOR		0.92(0.61–1.39)	0.685	1.00(0.71–1.41)	0.994	0.71(0.45–1.13)	0.148
**Categorical variables**				**Single drug resistance**		**Non-MDR two drug resistance**		**Multi-drug resistance (MDR)**	
**Variables**	**Categories**			**OR(95%Cl)**	***p***	**OR(95%Cl)**	***p***	**OR(95%Cl)**	***p***
Gender (ref: Male)	Female		UOR	0.82(0.34–1.97)	0.660	0.95(0.51–1.77)	0.885	0.80(0.33–1.90)	0.608
			AOR	1.02(0.37–2.86)	0.965	1.43(0.63–3.27)	0.391	1.01(0.35–2.96)	0.981
Age(ref:<20 yr)	20–45 yr		UOR	1.59(0.55–4.59)	0.390	2.22(1.07–4.62)	0.032	2.64(0.80–8.77)	0.111
			AOR	1.64(0.30–8.86)	0.563	**4.59(1.16–18.12)**	**0.030**	**7.30(1.10–48.59)**	**0.040**
	>45 yr		UOR	5.00(1.08–23.06)	0.039	4.73(1.38–16.19)	0.013	7.45(1.47–38.27)	0.015
			AOR	5.69(0.64–50.65)	0.119	**12.14(1.82–80.92)**	**0.010**	**30.01(2.70–333.06)**	**0.006**
Marital status (ref: Married)	Unmarried		UOR	0.68(0.27–1.71)	0.413	0.62(0.32–1.18)	0.144	0.57(0.22–1.47)	0.244
			AOR	1.71(0.38–7.67)	0.483	**4.16(1.20–14.48)**	**0.025**	4.83(0.99–23.66)	0.052
SES (ref: LIG)	MIG		UOR	0.81(0.27–2.39)	0.704	0.58(0.26–1.28)	0.176	0.77(0.26–2.26)	0.630
			AOR	0.47(0.13–1.67)	0.243	0.64(0.24–1.73)	0.382	0.92(0.25–3.35)	0.904
	HIG		UOR	–	–	–	–	–	–
			AOR	–	–	–	–	–	–
Resident of (ref: Kolkata)	North 24 Parganas		UOR	3.86(0.67–22.35)	0.131	1.24(0.25–6.15)	0.792	–	–
			AOR	5.37(0.69–41.42)	0.107	2.46(0.30–20.49)	0.405	–	–
	South 24 Parganas		UOR	3.48(1.07–11.33)	0.039	2.62(0.98–7.00)	0.054	1.47(0.4–5.44)	0.561
			AOR	**3.61(1.03–12.63)**	**0.044**	**3.54(1.14–10.91)**	**0.028**	1.93(0.45–8.16)	0.374
Sputum smear grading (ref: Single positive)	Double positive		UOR	2.94(1.26–6.83)	0.012	1.03(0.57–1.85)	0.920	1.32(0.60–2.92)	0.491
			AOR	2.52(0.87–7.35)	0.089	0.65(0.27–1.56)	0.338	0.90(0.29–2.83)	0.858
	Triple positive		UOR	2.83(0.36–22.39)	0.324	3.78(0.84–16.91)	0.082	1.70(0.22–13.02)	0.610
			AOR	2.10(0.18–25.19)	0.557	2.41(0.34–16.99)	0.377	0.85(0.06–11.27)	0.904
X-ray(ref: Minimal)	Moderately advanced		UOR	1.27(0.50–3.27)	0.616	0.76(0.39–1.46)	0.405	0.56(0.22–1.40)	0.216
			AOR	0.83(0.27–2.51)	0.738	**0.27(0.11–0.67)**	**0.004**	**0.16(0.05–0.53)**	**0.003**
	Far advanced		UOR	1.43(0.46–4.44)	0.534	0.67(0.29–1.53)	0.339	0.97(0.34–2.78)	0.958
			AOR	0.58(0.14–2.44)	0.455	**0.14(0.04–0.49)**	**0.002**	**0.14(0.03–0.68)**	**0.015**
	Cavity		UOR	2.54(0.48–13.46)	0.272	2.12(0.58–7.75)	0.257	0.78(0.12–5.18)	0.795
			AOR	0.78(0.11–5.96)	0.827	0.29(0.05–1.64)	0.161	0.11(0.01–1.17)	0.067
Patient type (ref: New Infection)	Relapsed		UOR	2.20(0.90–5.42)	0.084	9.97(4.83–20.58)	<.0001	9.67(3.35–27.91)	<.0001
			AOR	3.01(0.62–15.35)	0.168	**12.02(3.51–41.15)**	**<.0001**	**13.84(2.55–75.06)**	**0.002**
	Defaulter		UOR	1.15(0.25–5.24)	0.859	9.23(3.31–26.05)	<.0001	10.06(2.55–39.69)	0.001
			AOR	1.79(0.31–10.18)	**0.510**	**16.87(4.77–59.69)**	**<.0001**	**33.01(5.96–182.78)**	**<.0001**
	Treatment failure		UOR	1.91(0.11–32.00)	0.652	26.88(3.45–209.19)	0.002	50.28(5.42–465.91)	0.001
			AOR	1.67(0.05–59.93)	0.780	**31.86(2.66–381.62)**	**0.006**	**63.19(3.63–>99.99)**	**0.005**

SES = Socio-economic status; LIG = Low-income group; MIG = Middle-income group; HIG = High-income group; UOR = Unadjusted Odds ratio; AOR = Adjusted Odds ratio; OR = Odds ratio; CI = Confidence interval; *p* = *p* value.

“–” refers to cells for which due to inadequate number of observation valid statistical results (OR, CI, *p* value) could not be determined

## Discussion

Among 350 adolescent and adult smear-confirmed PTB patients due to M. tuberculosis infection, who attended two tertiary care hospitals in Kolkata between 2010–13, 68.57% belonged to relapse (44.00%), defaulter (15.43%) or treatment-failure (9.14%) categories, 64.00% had poorer X-ray features (moderately advanced = 37.71%, far advanced = 17.14% and cavities = 9.14%) and 12.29% were diagnosed with MDR-PTB. MDR-TB consisted 6.36% of total new infections cases, but 15% among the previously treated group. These findings probably serve as a grim reminder of the prevailing complex scenario of PTB in the largest metropolitan city of eastern India, which is not unlike rest of the country. [14], [23], [24] It is worth noting that the burden of MDR-PTB among newly infected [24] and previously treated cases [23] in most Indian states have consistently remained high during past two decades, and even more alarmingly, a recent report suggested a gradually increasing trend in MDR-PTB prevalence across the country. [14] In view of the above, our study findings convey that Kolkata is no exception.

In this study, corroborating with prior researches, having higher BMI was negatively associated with being a relapse/defaulter/treatment-failure case. [25], [26] Improved nutritional status, which can positively influence immunity and treatment outcome, could be cited as a possible explanation; however, such finding could also have been an artifact of reverse causation, as implicated by deterioration of general health among relapse/defaulter/treatment-failure cases. We believe, our results indicate the importance of providing nutritional improvement counseling/advices during the course of TB treatment.

Relapsed cases and defaulters were found to have, counter-intuitively, higher hemoglobin level. However, this again might be explained by possible reverse causation resulting from discontinuation of ATD therapy following initial physiological improvement (thus increase in hemoglobin) with the therapy. Thus, proper adherence counseling of patients at the time of initiating ATD is profoundly recommended.

Female subjects, in our study, were more likely to default in treatment compared to males, contradicting findings from previous studies, conducted elsewhere. [25], [27], [28] We hypothesize that the socio-cultural norms prevailing in the study area might have led to either lower awareness/adherence regarding ATD among females or had resulted in an underrepresentation of females among attendees of tertiary healthcare centers with uncomplicated, new infections.

Higher age was associated positively with relapse, potentially owing to poorer treatment outcomes resulting from different biological (diminishing immune function with increasing age) and social issues (self-neglect and poor knowledge/perception among elderly populace) working independently or in tandem. [29].

Although the employed multivariate model lacked sufficient statistical power, results of bivariate analyses suggested that poorer prognostic categories of sputum-smear and chest radiographic features were both associated with higher likelihood of being a relapse/treatment-failure case. Taking into account tubercular disease pathogenesis, these findings seem inherently plausible. In the current study, compared to the corresponding reference groups, females, elderly, unmarried and worse grades of sputum-smear were positively associated with poorer/more advanced chest-X-ray features, but higher BMI appeared to be a negative correlate of the same. Based on above, it is suggested that these socio-demographic and clinical parameters might well be taken into account while planning, designing and implementing preventive and curative components of the TB control program in this part of the country.

In this study higher age was found to be associated with higher odds of being MDR or non-MDR 2 drug resistant PTB cases, which highlighted the crucial role of poor awareness/perception, healthcare access/utilization and relatively lower social attention towards the elderly populace. [29].

Unmarried subjects also appeared to be more likely to be suffering from MDR or non-MDR 2 drug resistant PTB, compared to their married counterparts, and behavioral factors might be implicated behind this association. [30].

In sync with our observation regarding treatment outcomes, higher BMI seemed to be also associated negatively with emergence of drug resistance among participating PTB cases.

In corroboration with prior findings, relapse/defaulters/treatment-failure cases were found to have higher propensity of being MDR and non-MDR 2 drug resistant cases of PTB. [17], [31], [32].

Interestingly, it was observed that subjects with advanced radiological features had lower odds of having MDR or non-MDR 2 drug resistant PTB. Such observation points towards the possibility that cases with advanced radiological findings might be more likely to have been subjected to aggressive therapeutic and behavioral (counseling) interventions, often leading to improved treatment response and adherence, and therefore, lower likelihood of development of resistance.

Overall, the findings regarding resistance pattern seen in our study participants, make us infer that appropriate and intensive counseling of ATD initiates should be emphasized upon, in order to improve adherence to the treatment protocol among the PTB cases, and thereby reduce emergence of resistance.

Our study suffered from some major limitations. Due to the cross-sectional design, causal interpretations of the observed associations were not possible and temporal ambiguity affected inferences due to the potential for reverse causation. As the study was conducted in two tertiary care hospitals of Kolkata, often receiving referred (and relatively severe) cases from lower level healthcare providers, we might have overestimated the overall burden and some severity characteristics. Hence, extrapolation of the results beyond the study sample is not recommended, as lack of external validity may be suspected.

Despite of these limitations, we believe, our study findings have been bolstered by employing advanced laboratory techniques like MAS-PCR and sophisticated statistical analyses. The results of this study may help concerned policy makers to gain important insights into the aspects and correlates of drug resistance and treatment outcomes of PTB cases in this part of India, where similar efforts have been scanty till date.

## Conclusions

Implementation of targeted intervention and appropriate counseling seem to be an urgent requirement in order to improve the efficacy of various TB control approaches for specific demographics like females, elderly and unmarried in Kolkata.

## References

[1] World Health Organization (WHO) website. Global Tuberculosis Report, 2013. Geneva, Switzerland. Available: http://www.who.int/tb/publications/global_report/en/. Accessed 2014 May 15.

[2] World Health Organization (WHO) website. World TB Day 2013. Geneva, Switzerland. Available: http://www.searo.who.int/india/topics/tuberculosis/tbday_2013/en/. Accessed 2014 May 20.

[3] Agarwal S, Chauhan L (2005) Tuberculosis control in India: Directorate General of Health Services, Ministry of Health and Family Welfare, Govt. of India.

[4] Tuberculosis Control (TBC) website. Director General of Health Services, Ministry of Health and Family Welfare, Govt. of India. Available: http://www.tbcindia.nic.in/. Accessed 2014 May 22.

[5] Khatri GR, Frieden TR (2002) Controlling tuberculosis in India. N Engl J Med 347: 1420–1425. 12409545 10.1056/NEJMsa020098

[6] Sharma SK, Mohan A (2006) Multidrug-resistant tuberculosis: a menace that threatens to destabilize tuberculosis control. Chest 130: 261–272. 16840411 10.1378/chest.130.1.261

[7] Chowdhury IH, Sen A, Bahar B, Hazra A, Chakraborty U, et al (2012) A molecular approach to identification and profiling of first-line-drug-resistant mycobacteria from sputum of pulmonary tuberculosis patients. J Clin Microbiol 50: 2082–2084. 22461679 10.1128/JCM.06093-11 PMC3372138

[8] Patel D, Madan I (2000) Methicillin-resistant Staphylococcus aureus and multidrug resistant tuberculosis: Part 2. Occup Med (Lond) 50: 395–397. 10994241 10.1093/occmed/50.6.395

[9] Faustini A, Hall AJ, Perucci CA (2006) Risk factors for multidrug resistant tuberculosis in Europe: a systematic review. Thorax 61: 158–163. 16254056 10.1136/thx.2005.045963 PMC2104570

[10] Paramasivan CN, Venkataraman P (2004) Drug resistance in tuberculosis in India. Indian J Med Res 120: 377–386. 15520487

[11] Snider DE Jr, Kelly GD, Cauthen GM, Thompson NJ, Kilburn JO (1985) Infection and disease among contacts of tuberculosis cases with drug-resistant and drug-susceptible bacilli. Am Rev Respir Dis 132: 125–132. 3925826 10.1164/arrd.1985.132.1.125

[12] Ahuja SD, Ashkin D, Avendano M, Banerjee R, Bauer M, et al (2012) Multidrug resistant pulmonary tuberculosis treatment regimens and patient outcomes: an individual patient data meta-analysis of 9,153 patients. PLoS Med 9: e1001300. 22952439 10.1371/journal.pmed.1001300 PMC3429397

[13] Della-Latta P (2007) Mycobacteriology and Antimycobacterial Susceptibility Testing. In: Isenberg HD, Garcia LS, editors. Clinical Microbiology Procedures Handbook. 2nd ed: ASM Press. Washington DC, USA. pp. 7.0.1–7.3.3.

[14] Maurya AK, Singh AK, Kumar M, Umrao J, Kant S, et al (2013) Changing patterns and trends of multidrug-resistant tuberculosis at referral centre in Northern India: a 4-year experience. Indian J Med Microbiol 31: 40–46. 23508428 10.4103/0255-0857.108720

[15] Leitch AG (2002) Pulmonary tuberculosis: clinical features. In: Anthony Seaton DS, A. Gordon Leitch, editor. Crofton and Douglas's Respiratory Diseases, Fifth ed: Oxford, UK: Blackwell Science Ltd. pp. 507–527.

[16] Chakraborty U, Goswami A, Saha S, Mukherjee T, Dey SK, et al (2013) Tumour necrosis factor-alpha and nitric oxide response in different categories of tuberculosis patients. Int J Tuberc Lung Dis 17: 505–510. 23485383 10.5588/ijtld.12.0196

[17] Weyer K, Brand J, Lancaster J, Levin J, Van der Walt M (2007) Determinants of multidrug-resistant tuberculosis in South Africa: results from a national survey. S Afr Med J 97: 1120–1128. 18250922

[18] Kent P, Kubica G (1985) A guide for the level III laboratory. Public health mycobacteriology Atlanta, GA: Centers for Disease Control, US Department of Health and Human Services.

[19] Hosek J, Svastova P, Moravkova M, Pavlik I, Bartos M (2006) Methods of mycobacterial DNA isolation from different biological material: a review. Vet Med (Praha) 51: 180–192.

[20] Bhattacharya B, Karak K, Ghosal AG, Roy A, Das S, et al (2003) Development of a new sensitive and efficient multiplex polymerase chain reaction (PCR) for identification and differentiation of different mycobacterial species. Trop Med Int Health 8: 150–157. 12581441 10.1046/j.1365-3156.2003.01007.x

[21] Yang Z, Durmaz R, Yang D, Gunal S, Zhang L, et al (2005) Simultaneous detection of isoniazid, rifampin, and ethambutol resistance of Mycobacterium tuberculosis by a single multiplex allele-specific polymerase chain reaction (PCR) assay. Diagn Microbiol Infect Dis 53: 201–208. 16243477 10.1016/j.diagmicrobio.2005.06.007

[22] Kwak C, Clayton-Matthews A (2002) Multinomial logistic regression. Nurs Res 51: 404–410. 12464761 10.1097/00006199-200211000-00009

[23] Sharma SK, Kumar S, Saha P, George N, Arora S, et al (2011) Prevalence of multidrug-resistant tuberculosis among category II pulmonary tuberculosis patients. Indian J Med Res 133: 312–315. 21441686 PMC3103157

[24] Sharma SK, Kaushik G, Jha B, George N, Arora S, et al (2011) Prevalence of multidrug-resistant tuberculosis among newly diagnosed cases of sputum-positive pulmonary tuberculosis. Indian J Med Res 133: 308–311. 21441685 PMC3103156

[25] Dooley KE, Lahlou O, Ghali I, Knudsen J, Elmessaoudi MD, et al (2011) Risk factors for tuberculosis treatment failure, default, or relapse and outcomes of retreatment in Morocco. BMC Public Health 11: 140. 21356062 10.1186/1471-2458-11-140 PMC3053250

[26] Khan A, Sterling TR, Reves R, Vernon A, Horsburgh CR (2006) Lack of weight gain and relapse risk in a large tuberculosis treatment trial. Am J Respir Crit Care Med 174: 344–348. 16709935 10.1164/rccm.200511-1834OC

[27] Santha T, Garg R, Frieden T, Chandrasekaran V, Subramani R, et al (2002) Risk factors associated with default, failure and death among tuberculosis patients treated in a DOTS programme in Tiruvallur District, South India, 2000. Int J Tuberc Lung Dis 6: 780–788. 12234133

[28] Jha UM, Satyanarayana S, Dewan PK, Chadha S, Wares F, et al (2010) Risk factors for treatment default among re-treatment tuberculosis patients in India, 2006. PLoS One 5: e8873. 20111727 10.1371/journal.pone.0008873 PMC2810342

[29] Zaveri H, Mansuri S, Patel V (2010) Use of potentially inappropriate medicines in elderly: A prospective study in medicine out-patient department of a tertiary care teaching hospital. Indian journal of Pharmacology 42: 95. 20711374 10.4103/0253-7613.64499 PMC2907023

[30] Young JT (2004) Health in the Developing World: Health Status and Healthcare Utilization in Matlab, Bangladesh: University of Colorado.

[31] Banu S, Mahmud AM, Rahman MT, Hossain A, Uddin MKM, et al (2012) Multidrug-resistant tuberculosis in admitted patients at a tertiary referral hospital of Bangladesh. PLoS One 7: e40545. 22808189 10.1371/journal.pone.0040545 PMC3394739

[32] Lomtadze N, Aspindzelashvili R, Janjgava M, Mirtskhulava V, Wright A, et al (2009) Prevalence and risk factors for multidrug-resistant tuberculosis in Republic of Georgia: a population based study. Int J Tuberc Lung Dis 13: 68–73. 19105881 PMC2645031

